# Accelerating Quantum Decay by Multiple Tunneling Barriers

**DOI:** 10.3390/e25091345

**Published:** 2023-09-16

**Authors:** Ermanno Pinotti, Stefano Longhi

**Affiliations:** 1 Dipartimento di Fisica, Politecnico di Milano, Piazza L. da Vinci 32, I-20133 Milano, Italy; 2 IFISC (UIB-CSIC), Instituto de Fisica Interdisciplinar y Sistemas Complejos, E-07122 Palma de Mallorca, Spain

**Keywords:** quantum tunneling, quasi-bound states, tight binding lattices

## Abstract

A quantum particle constrained between two high potential barriers provides a paradigmatic example of a system sustaining quasi-bound (or resonance) states. When the system is prepared in one of such quasi-bound states, the wave function approximately maintains its shape but decays in time in a nearly exponential manner radiating into the surrounding space, the lifetime being of the order of the reciprocal of the width of the resonance peak in the transmission spectrum. Naively, one could think that adding more lateral barriers would preferentially slow down or prevent the quantum decay since tunneling is expected to become less probable and due to quantum backflow induced by multiple scattering processes. However, this is not always the case and in the early stage of the dynamics quantum decay can be accelerated (rather than decelerated) by additional lateral barriers, even when the barrier heights are arbitrarily large. The decay acceleration originates from resonant tunneling effects and is associated to large deviations from an exponential decay law. We discuss such a counterintuitive phenomenon by considering the hopping dynamics of a quantum particle on a tight-binding lattice with on-site potential barriers.

## 1. Introduction

**Figure 1:**
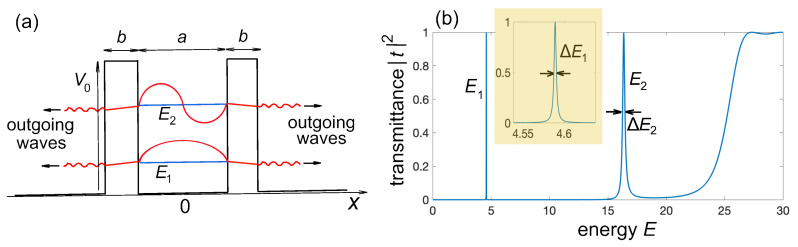
(**a**) Schematic of a double rectangular potential barrier sustaining resonance (quasi-bound) states at energies $E=E_{1},E_{2},\ldots$. Barrier height is $V_{0}$, barrier width is *b*, and barrier distance is d=a+b. (**b**) Spectral transmittance ${\left|t\left(E\right)\right|}^{2}$ of the two-barrier potential versus energy of the incidence wave. Parameter values are a=b=1, $V_{0}=20$. The inset in (**b**) shows an enlargement of the first resonance at energy $E=E_{1}$, which is well approximated by a Lorentzian curve (Breit–Wigner resonance). The two resonance peaks in (**b**) correspond to the quasi-bound states depicted in (**a**) by the solid red curves. In the high potential barrier limit, the quasi-bound state can be approximately written as in Equation (6), where θ(x,t) describes the small-amplitude outgoing waves escaping from the barrier region, owing to evanescent tunneling (oscillating tails in the plots) and τ=1/ΔE is the lifetime, which is the inverse of the width ΔE of the corresponding resonance peak in (**b**).

Quantum tunneling is ubiquitous in quantum mechanics where a particle has a non-zero probability of passing through a classically forbidden energy barrier, even though it does not have enough energy to overcome that barrier according to classical physics [1,2]. This behavior arises from the wave-like nature of particles at the quantum level, and can be thus also observed for classical waves such as light and sound waves (see, e.g., [3,4,5,6]). One of the main predictions of quantum tunneling is the instability and decay of a quantum particle trapped by potential barriers of finite heights, a prototypal example being α-decay in nuclear physics [7,8]. Perhaps the simplest one-dimensional quantum mechanical model possessing quasi-stationary (resonance) states, decaying via tunneling leakage, is the double rectangular potential barrier model [Figure 1a], which was introduced in a famous paper by Gamov to model α decay [7]. When the barrier height $V_{0}$ is infinite, the system sustains a set of stationary (non-decaying) bound states at some quantized energies; however, when the barrier height $V_{0}$ is not infinite, some of these states, those with energies close to the bottom of the barriers, become metastable, i.e., they become resonance states (also known as Gamow or Siegert states, or quasi-bound states; see, e.g., [9,10,11,12,13,14] and references therein). This means that an initial wave function prepared in a bound state of the infinite barrier approximately maintains its shape but decays in time in a nearly exponential manner through tunneling leakage across the barriers, generating small-amplitude outgoing waves that spread outward the barrier region [9,10,11]. The signature of resonance states are the characteristic Breit–Wigner resonance peaks in the transmission spectrum of the double potential barrier, and the lifetimes of the resonance states are of the order of the reciprocal of the widths of the Breit–Wigner resonances [11] [see Figure 1b].

The quantum decay does not strictly follow a simple exponential decay law, and deviations from an exponential decay universally arise in the short and long time scales [15,16,17,18,19,20], leading to Zeno-like dynamics, i.e., the deceleration (Zeno effect) or the acceleration (anti-Zeno effect) of the decay by frequent observations of the system (see, e.g., [21,22,23,24,25] and references therein). Strong deviations from an exponential decay law are generally observed due to interference between different decay pathways, strong coupling with a featureless bath, or with an engineered bath, which introduce memory effects and non-Markovian behavior, or in the presence of edge effects or localized states, such as in disordered systems, leading to revivals and limited quantum decay [26,27,28].

Quantum leakage dynamics in the double-barrier potential is clearly modified when lateral barriers are added. Such additional barriers introduce interference effects and make the quantum decay greatly non-exponential rather generally. Naively, one could think that additional barriers would preferentially slow down the decay, since the tunneling is expected to become less probable and because of the back flow into the original excitation region. For example, for stochastic barriers one expects Anderson localization [29,30], leading to highly non-Markovian dynamics, Rabi-like oscillations, and limited quantum decay [27,28,31]. However, this picture may fail in other cases as multiple interference effects could play in a reversed way.

In this work, we unveil the rather counterintuitive effect of quantum decay acceleration of a resonance state in the double-barrier model induced by additional later barriers: rather than slowing down the decay, they can greatly accelerate the quantum decay, even when the heights of the barriers are unbounded. This unusual phenomenon is explained in terms of resonant tunneling (hopping) and studied by considering in details the decay of resonance states in potential barriers on a tight-binding lattice, which can be emulated in photonic settings using evanescently coupled optical waveguide lattices [32,33,34] or grating structures [35,36,37].

## 2. Acceleration and Deceleration of Quantum Decay in the Double-Barrier Model: Some Preliminary Considerations

Before considering quantum decay in tight-binding models with on-site potential barriers, it is worth presenting some preliminary results and discussion on the decay dynamics of resonance states in Gamov’s model for the continuous Schrödinger equation in one spatial dimension, which is written in dimensionless units as

(1) $$i\frac{\partial \psi }{\partial t}=-\frac{\partial^{2}\psi }{\partial x^{2}}+V\left(x\right)\psi$$

where ψ=ψ(x,t) is the wave function and V(x) is the potential. Let us first assume that V(x) describes a double rectangular barrier, with barrier height $V_{0}$, barrier width *b*, and barrier distance d=b+a [Figure 1a]. Figure 1b shows a typical behavior of spectral transmittance ${\left|t\left(E\right)\right|}^{2}$ versus energy *E* of the incidence plane wave. The transmission amplitude t(E) can be calculated by standard textbook methods and reads

(2) $$t\left(E\right)=\frac{t_{1}^{2}\exp\left(ik_{0}a\right)}{1-r_{1}^{2}\exp\left(2ik_{0}a\right)}$$

where $r_{1}\left(E\right)$ and $t_{1}\left(E\right)$ are the reflection and transmission amplitudes of the single barrier, given by

(3) $$\begin{array}{ccc} t_{1}\left(E\right) & = & \frac{4k_{0}k_{1}\exp\left(ik_{1}b\right)}{{\left(k_{0}+k_{1}\right)}^{2}-{\left(k_{0}-k_{1}\right)}^{2}\exp\left(2ik_{1}b\right)} \end{array}$$

(4) $$\begin{array}{ccc} r_{1}\left(E\right) & = & \frac{\left(k_{0}^{2}-k_{1}^{2}\right)\sin\left(k_{1}b\right)}{\left(k_{0}^{2}+k_{1}^{2}\right)\sin\left(k_{1}b\right)+2ik_{0}k_{1}\cos\left(k_{1}b\right)} \end{array}$$

and

(5) $$k_{0}\equiv \sqrt{E}\;,\;\;\;k_{1}\equiv \sqrt{E-V_{0}}.$$

The spectral transmittance clearly shows resonance peaks at some energies (two peaks at energies $E=E_{1},E_{2}$ in the plot of Figure 1b), which correspond to quasi-bound states. In the high-barrier limit, i.e., very narrow resonances (such as the first resonance at $E=E_{1}$ shown in the inset of Figure 1b), the resonance curve is Lorentzian-shaped to a high degree of approximation (Breit–Wigner resonance) and the corresponding quasi-bound state can be roughly speaking written as

**Figure 2:**
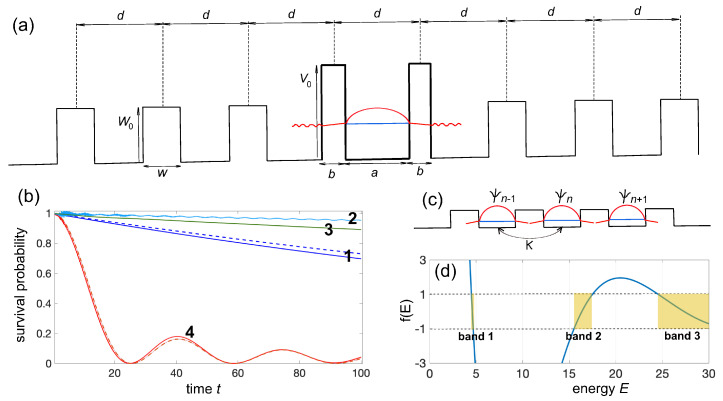
(**a**) Schematic of a quasi-bound state in the double-barrier model (bold solid curve) that radiates in space with additional later barriers (thin solid curves). All barriers are equally spaced by a distance *d*. The size *w* and height $W_{0}$ of the lateral barriers can differ from those of the two central barriers. (**b**) Numerically computed decay behavior of the survival probability $P\left(t\right)=\int_{-a/2}^{a/2}dx{\left|\psi \left(x,t\right)\right|}^{2}$, normalized to its initial value P(0), for $V_{0}=20$, a=b=1, d=2, and for a few different values of *w* and $W_{0}$. Curve 1: quasi-bound state radiating in free space ($W_{0}=0$); the dashed curve is the exponential decay law with lifetime $\tau =1/\Delta E_{1}≃322.6$ predicted by the width of the first resonance peak in the spectrum of Figure 1b. Curve 2: $W_{0}=V_{0}=20$, w=b/2=0.5. Curve 3: $W_{0}=V_{0}/5=4$, w=b/2=0.5. Curve 4: $W_{0}=V_{0}=20$, w=b=1; the dashed curve 4 is the decay behavior $P\left(t\right)=|J_{0}{\left(2\kappa t\right)|}^{2}$ predicted by the tight-binding analysis of resonant tunneling [Equation (10)]. (**c**) The Kronig-Penney model for $W_{0}=V_{0}$ and w=b. The set of resonant quasi-bound states trapped in adjacent double-barrier potentials form an energy band, and excitation can hop between adjacent cells of the crystal with a hopping rate κ. (**d**) Geometrical construction of the energy bands for the Kronig-Penney model. The solid curve shows the behavior of the function f(E) versus energy *E*; the function f(E) is defined by Equation (8) in the main text. The allowed energy bands are determined by the inequality |f(E)|≤1 and are indicated as band 1, band 2, and band 3 in the figure. The narrow band 1 arises from the hybridization of the lowest-energy resonant quasi-bound states sustained by each double potential barrier (unit cell) in the crystal.

(6) ψ(x,t)=ψ(x,0)exp(−iEt−t/2τ)+θ(x,t)

where ψ(x,0), *E* are close to the bound state wave function and corresponding (possibly shifted) eigenenergy in the infinite $V_{0}=\infty$ limit, τ=1/ΔE is the lifetime of the quasi-bound state, ΔE is the full-width at half-maximum of the Breit–Wigner resonance, and θ(x,t) describes the small-amplitude outgoing waves in the outer regions of the barriers (see Figure 1a). An example of a nearly exponential decay of the lowest resonance state is shown in Figure 2b, which depicts the decay behavior of the survival probability to find the particle between the two barriers,

(7) $$P\left(t\right)=\int_{-a/2}^{a/2}dx{\left|\psi \left(x,t\right)\right|}^{2},$$

normalized to its initial value P(0). Here, ψ(x,0) is assumed to be close to the lowest-energy bound state of the same barrier model but with $V_{0}=\infty$, i.e., ψ(x,0)∝cos(πx/a) for |x|<a/2 and ψ(x,0)=0, otherwise propagated for a short time interval (Δt=3) to remove fast transient oscillations in the behavior of P(t). The results are obtained by numerical integration of the time-dependent Schrödinger Equation (1) using an accurate pseudospectral split-step method. The decay dynamics (solid curve 1 in Figure 2b) is rather well fitted by an exponential curve (dashed curve 1 in Figure 2b) with a lifetime close to the theoretical value $\tau =1/\Delta E_{1}≃322.6$ predicted from the spectral width $\Delta E_{1}≃0.0031$ of the lowest Breit–Wigner resonance peak. A similar behavior is found when the system is initially prepared in the second resonance state, i.e., ψ(x,0)∝sin(2πx/a) for |x|<a/2 and ψ(x,0)=0 otherwise, the exponential decay displaying a much shorter lifetime ($\tau =1/\Delta E_{2}≃4.50$), according to the larger width $\Delta E_{2}$ of the second resonance peak in Figure 1b.

Clearly, the decay dynamics is greatly modified and can largely deviate from an exponential law when we consider additional lateral barriers, because the outgoing waves that escape via tunneling from the two barriers can be back-reflected and re-injected into the original spatial region |x|<a/2. The final decay law P(t) is the result of a complex multiple-interference process which, depending on the choice of the additional barriers, can either decelerate or accelerate the decay. The fact that additional barriers can slow down the decay of the survival probability is not surprising; however, it is more elusive regarding how and why the decay can be accelerated in some cases. One of the main mechanisms that explains decay acceleration is *resonant tunneling* (see e.g., [38]). This point can be illustrated by considering, as an example, the case of an array of equally spaced barriers; see Figure 2a. Besides the two barriers seen in Figure 1a, we now add a sequence of equally spaced barriers of height $W_{0}$, same space separation d=a+b, and barrier width *w*. Barrier height $W_{0}$ and width *w* can be rather generally different than $V_{0}$ and *a*. Curves 2 and 3 in Figure 2b show the decay dynamics of the survival probability when either or both *w* and $W_{0}$ differ from *b* and $V_{0}$, clearly indicating that the additional barriers decelerate the decay. However, a striking effect is observed when w=b and $W_{0}=V_{0}$: the decay of survival probability is much faster and the decay dynamics greatly deviates from an exponential law (curve 4 in Figure 2b). The decay acceleration can be explained on the basis of resonant tunneling (hopping) between resonant quasi-bound states that are sustained by adjacent double-potential barriers, as schematically shown in Figure 2c. In fact, for w=b and $W_{0}=V_{0}$, the potential V(x) is strictly periodic with period d=a+b, and such a periodic potential corresponds to the well-known Kronig–Penney model in solid-state physics [39,40]. Basically, the various resonant quasi-bound states sustained in adjacent double-barriers hybridize and give rise to a set of bands. The dispersion curves E=E(k) of the various bands are defined implicitly by the relation (see e.g., [40])

cos(kd)=f(E)

where we have set

(8) $$f\left(E\right)=\cos\left(a\sqrt{E}\right)\cos\left(b\sqrt{E-V_{0}}\right)-\frac{2E-V_{0}}{2\sqrt{E(E-V_{0}})}\sin\left(a\sqrt{E}\right)\sin\left(b\sqrt{E-V_{0}}\right).$$

In the above equation, *k* is the Bloch wave number, which varies in the first Brillouin zone −π/d<k≤π/d. The band dispersion curves, defined by the relation cos(kd)=f(E), can be solved graphically, as shown in Figure 2d. The low-energy narrow-band in Figure 2d, indicated as band 1 and centered at around $E=E_{1}≃4.589$, arises from the weak overlapping (hybridization) of resonant quasi-bound states with energies $E_{1}$ in adjacent unit cells of the crystal, and its bandwidth 4κ≃0.1884 defines the hopping amplitude κ between adjacent sites within a tight-binding description. In the nearest-neighbor approximation, an initial excitation of one of such quasi-bound mode can jump from one unit cell to its neighbor in either direction with a rate κ, and the spreading dynamics is ballistic and governed by the set of coupled equations (see e.g., [40,41,42] )

(9) $$i\frac{d\psi_{n}}{dt}=-\kappa \left(\psi_{n+1}+\psi_{n-1}\right)$$

where $\psi_{n}$ is the amplitude of the quasi-bound state at the *n*-th unit cell. The decay behavior of the survival probability is then given analytically in terms of $J_{0}$ Bessel function, namely [41]

(10) $$P\left(t\right)=|J_{0}{\left(2\kappa t\right)|}^{2}.$$

The solid curve 4 in Figure 2b shows the numerically computed behavior of P(t) for $V_{0}=W_{0}=20$ and a=b=w=1, which is very well fitted by the theoretical prediction given by Equation (10) (dashed curve 4), in which the hopping rate κ≃0.0471 is estimated from the width of the narrow band of Figure 2d. Clearly, the decay of survival probability greatly deviates from an exponential curve and, in the early stage, it is much faster than other cases (curves 1–3 in Figure 2b), where resonant tunneling is prevented: the hopping dynamics enabled by resonant tunneling makes the decay faster.

## 3. Decay Acceleration by Resonant Tunneling in Tight-Binding Lattices

**Figure 3:**
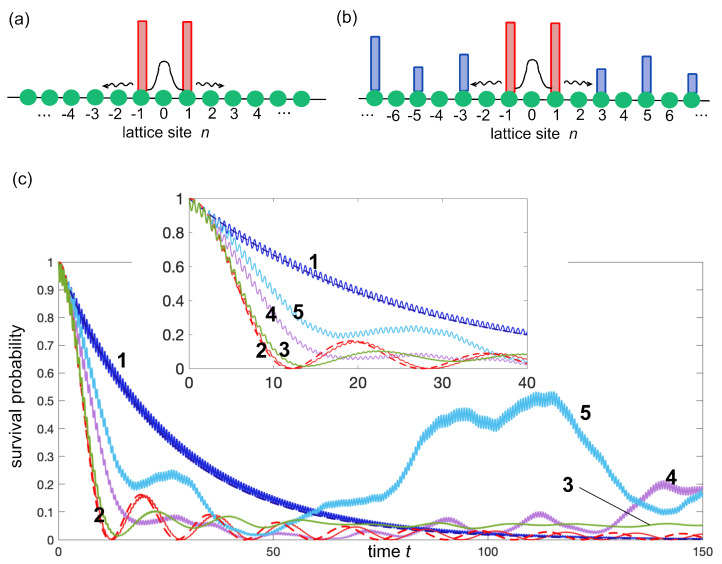
(**a**) Schematic of a double-barrier potential on a tight-binding lattice. A quasi-bound state trapped between the two high barriers radiates into the lattice. (**b**) The multi-barrier model. Additional potential barriers are introduced at odd lattice sites. (**c**) Decay of the survival probability $P\left(t\right)=|\psi_{0}{\left(t\right)|}^{2}$ for a few different settings of potential barriers and for κ=1, $V_{0}=10$. Curve 1 is the nearly exponential decay behavior of the double-barrier model of panel (**a**), i.e., in the absence of additional lateral barriers. The almost overlapped dashed curve is the exponential decay law predicted by Equation (16). Curve 2 is the decay curve obtained for $W_{n}=V_{0}=10$, and the almost overlapped dashed curve is the theoretical prediction given by Equation (24). Curve 3 is the decay behavior for the Bernoulli model with $V_{1}=V_{0}$, $V_{2}=V_{0}/2$, and p=0.5. Curve 4 is the decay behavior corresponding to the symmetric Stark potential barrier model with F=1. Curve 5 is the decay behavior for the parabolic potential barrier model with F=0.1. The inset in (**c**) shows an enlargement of the decay dynamics in the early stage.

The phenomenon of decay acceleration in the early stage of the dynamics mediated by hopping between adjacent resonant quasi-bound states, discussed in the previous section, suggests to re-examine quantum decay and tunneling effects in the framework of simple tight-binding models [32,43,44]. Such models, besides being simpler to study and simulate, can be readily implemented in photonic settings using engineered arrays of evanescently coupled optical waveguides. In fact, they have served over the past two decades as feasible laboratory tools for the observation of non-exponential decay features and Zeno dynamics with photons [5,33,34,45,46,47]. The simplest two-barrier system sustaining one resonance state on a tight-binding lattice is described by the Hamiltonian [Figure 3a]

(11) $$H=-\kappa \sum_{n}(|n\rangle \langle n+1|+|n+1\rangle \langle n|)+V_{0}\sum_{n=\pm 1}\left|n\rangle \langle n\right|$$

where κ is the hopping rate between adjacent sites of the lattice and $V_{0}$ is the on-site potential barrier at the two sites n=±1. For the sake of definiteness, we assume $V_{0}>0$; however, on a lattice a quasi-bound state is also sustained for $V_{0}<0$. Indicating by $\psi_{n}$ the wave amplitude at the *n*-th lattice site, i.e., after letting $|\psi \left(t\right)\rangle =\sum_{n}\psi_{n}\left(t\right)|n\rangle$, the Schrödinger equation $i\partial_{t}|\psi \left(t\right)\rangle =H|\psi \left(t\right)\rangle$ yields the set of coupled equations

(12) $$i\frac{d\psi_{n}}{dt}=-\kappa \left(\psi_{n+1}+\psi_{n-1}\right)+V_{0}\left(\psi_{1}+\psi_{-1}\right).$$

In the high-barrier limit $V_{0}\gg \kappa$, an initial excitation at time t=0 of site n=0, trapped between the two high potential barriers, is metastable and the survival probability, $P\left(t\right)=|\psi_{0}{\left(t\right)|}^{2}$, decays in time nearly exponentially, as observed in numerical simulations of Equation (12) assuming the initial conditions $\psi_{n}\left(0\right)=\delta_{n,0}$; see curve 1 in Figure 3c. Note that a small-amplitude and fast oscillation is superimposed to the exponential decay, the amplitude of the oscillations vanishing in the $V_{0}/\kappa \to \infty$ limit. The lifetime of the quasi-bound state at site n=0 can be readily estimated by adiabatic elimination from the dynamics of the small amplitudes at the sites $\psi_{\pm 1}$. In fact, in the high barrier limit $V_{0}/\kappa \gg 1$ one can assume in Equation (12) $|\left(d\psi_{1,-1}/dt\right)|\ll V_{0}\left|\psi_{1,-1}\right|$, and thus

(13) $$\psi_{1}≃\frac{\kappa }{V_{0}}\left(\psi_{0}+\psi_{2}\right)\;,\;\;\;\psi_{-1}≃\frac{\kappa }{V_{0}}\left(\psi_{0}+\psi_{-2}\right).$$

Taking into account for symmetry reasons that $\psi_{-n}\left(t\right)=\psi_{n}\left(t\right)$, after letting $\varphi_{0}\left(t\right)=\psi_{0}\left(t\right)\exp\left(i\Omega t\right)$ and $c_{n}\left(t\right)=\sqrt{2}\psi_{n+1}\left(t\right)\exp\left(i\Omega t\right)$ for n≥1, from Equations (12) and (13) one obtains

(14) $$\begin{array}{ccc} i\frac{d\varphi_{0}}{dt} & = & \Omega \varphi_{0}-\kappa_{1}c_{1}\\ i\frac{dc_{1}}{dt} & = & -\kappa_{1}\varphi_{0}-\kappa c_{2}\\ i\frac{dc_{n}}{dt} & = & -\kappa \left(c_{n+1}+c_{n-1}\right)\;\;\;\left(n\geq 2\right) \end{array}$$

where we have set

(15) $$\kappa_{1}\equiv \frac{\sqrt{2}\kappa^{2}}{V_{0}}\;,\;\;\Omega \equiv -\frac{\kappa^{2}}{V_{0}}.$$

The reduced model (14) can be cast in the standard Friedrichs–Lee (or Fano–Anderson) model, describing the decay of a single bound state weakly coupled to a featureless tight-binding continuum (see e.g., [34,48,49]), and in the Markovian approximation the survival probability can be calculated as

(16) $$P\left(t\right)=|\varphi_{0}{\left(t\right)|}^{2}≃\exp\left(-t/\tau \right)$$

where the lifetime τ is given by (see Appendix A for details)

(17) $$\tau =\frac{\kappa }{2\kappa_{1}^{2}}=\frac{V_{0}^{2}}{4\kappa^{3}}.$$

The exponential decay predicted by Equations (16) and (17) turns out to be in good agreement with the exact decay behavior found by numerical simulations (compare solid and dashed curves 1 in the inset of Figure 3c).

When lateral barriers are introduced, the decay dynamics is rather generally modified and does not follow anymore the exponential law from Equation (16). In order to observe decay acceleration by resonant tunneling, as suggested in Section 2 above, the potential barriers are added at odd potential sites solely; see Figure 3b. The Hamiltonian of the system reads

(18) $$H=-\kappa \sum_{n}(|n\rangle \langle n+1|+|n+1\rangle \langle n|)+\sum_{n}W_{n}\left|n\rangle \langle n\right|$$

where $W_{n}$ is the strength of the potential barrier at odd lattice sites, with $W_{\pm 1}=V_{0}$ and $W_{n}=0$ for *n* even. We mention that, in photonics, the tight-binding model (18) can be implemented using arrays of evanescently coupled optical waveguides, in which a uniform coupling constant κ and engineered propagation constant shifts $W_{n}$ are realized by judicious design of waveguide widths and spacing. For example, a linear gradient potential was realized in semiconductor waveguide arrays to demonstrate optical Bloch oscillations in Ref. [50].

After adiabatic elimination of the small amplitudes $\psi_{\pm 1}$ as discussed above [Equation (13)], the decay dynamics of $\psi_{0}\left(t\right)$ could be framed in the form of a single-level Fano–Anderson model, wherein the additional barriers $W_{n}$ clearly structure the continuum of states into which the state |0〉 is coupled, and could induce localization phenomena responsible for strong backflow and revivals in the dynamics [26,27,28]. It is precisely these effects that make decay acceleration possible in the early stage of the dynamics. The canonical Fano–Anderson form describing the decay process for the Hamiltonian (18) is detailed in the Appendix A, which derives the general form of the memory function entering in the integro-differential equation describing the decay dynamics of the amplitude $\psi_{0}\left(t\right)$. The memory function basically includes all the multiple reflection phenomena and delay effects arising from wave scattering of additional lateral barriers, which make the decay strongly non-Markovian. The form of the memory function depends in a complex way on the eigenstates of the bath Hamiltonian, and even if its form may be calculated analytically in very special cases [51], it is hard to provide general insights into the decay dynamics as governed by the integro-differential equation. However, for our purposes, we do not need to resort to the canonical Fano–Anderson model and in the following analysis we will provide some direct examples of quantum decay acceleration adopting the full Hamiltonian (18). To this aim, it is worth noting that the system described by Equation (18) is bipartite, and thus one can write the wave function as $|\psi \left(t\right)\rangle =\sum_{n}(a_{n}\left(t\right)|2n\rangle +b_{n}\left(t\right)|2n+1\rangle )$. The evolution equations for the wave amplitudes $a_{n}$ and $b_{n}$ at even and odd lattice sites read

(19) $$\begin{array}{ccc} i\frac{da_{n}}{dt} & = & -\kappa (b_{n-1}+b_{n}) \end{array}$$

(20) $$\begin{array}{ccc} i\frac{db_{n}}{dt} & = & -\kappa \left(a_{n}+a_{n+1}\right)+W_{2n+1}b_{n} \end{array}$$

which should be solved with the initial condition $a_{n}\left(0\right)=\delta_{n,0}$ and $b_{n}\left(0\right)=0$.

Let us now discuss a few prototypal examples of decay acceleration, observed in the early stage of the dynamics, induced by the additional lateral barriers.

(i) The first example of decay acceleration is obtained by assuming $W_{n}=V_{0}$ for *n* odd, which is the discrete analogue of the Kronig–Penney model considered in the previous section [Figure 2c]. In this case, the Hamiltonian (18) describes a bipartite lattice sustaining two bands. In the high barrier limit $V_{0}\gg \kappa$, to calculate the decay of the survival probability, we can adiabatically eliminate the small amplitudes $b_{n}$ from the dynamics by letting

(21) $$b_{n}≃\frac{\kappa }{V_{0}}\left(a_{n}+a_{n+1}\right)$$

so that from Equation (19) one obtains

(22) $$i\frac{da_{n}}{dt}=-\kappa_{1}\left(a_{n+1}+a_{n-1}+2a_{n}\right)$$

where we have set

(23) $$\kappa_{1}\equiv \frac{\kappa^{2}}{V_{0}}$$

The solution to Equation (22) with the initial condition $a_{n}\left(0\right)=\delta_{n,0}$ is given in terms of Bessel $J_{0}$ function [41,42] and the corresponding decay behavior of the survival probability reads

(24) $$P\left(t\right)=|a_{0}{\left(t\right)|}^{2}=J_{0}^{2}\left(2\kappa_{1}t\right).$$

Curve 2 in Figure 3c shows the numerically computed decay behavior of P(t) for $V_{0}/\kappa =10$, which turns out to be rather well fitted by the theoretical prediction given by Equation (24). Clearly, the decay largely deviates from an exponential law and, most importantly, it is accelerated as compared to curve 1, at least in the early-to-intermediate time scale of the decay.

(ii) As a second example of decay acceleration, let us assume that at odd sites *n*, with n≠±1, the potential $W_{n}$ can take two possible values, either $W_{n}=V_{1}$ or $W_{n}=V_{2}$, with probabilities *p* and 1−p, respectively (Bernoulli model [52]). Curve 3 in Figure 3c shows the numerically computed decay behavior of the survival probability for $V_{1}=V_{0}$, $V_{2}=V_{0}/2$, $V_{0}/\kappa =10$ and p=1/2, averaged over 200 realizations. Also, in this case, one can clearly observe an acceleration of the decay in the early stage of the dynamics, in spite of Anderson localization being able to take place in this model (see Appendix B). This means that, unlike the previous example (i), at long times the decay is not complete.

(iii) The third example of decay acceleration concerns deterministic potential barriers with continuously increasing and unbounded heights, namely we assume symmetric Stark potential barriers with $W_{-n}=W_{n}$ and

(25) $$W_{n}=\left\{\begin{array}{cc} F(n-1) & n\geq 1,\;\;n\;\mathrm{odd}\\ 0 & n\;\mathrm{even}. \end{array}\right.$$

Curve 4 in Figure 3c shows the numerically computed behavior of the survival probability $P\left(t\right)=|a_{0}{\left(t\right)|}^{2}$ for $V_{0}/\kappa =10$ and F=1, clearly showing the acceleration of the decay in the early stage of the decay. This is a rather striking and unexpected result, given that the added barriers have a monotonously increasing and unbounded height and the corresponding Hamiltonian (18) has an almost pure point spectrum with localized eigenstates (see Appendix B and [53]).

(iv) The fourth example of decay acceleration is analogous to the previous case, but with a quadratic (rather than linear) increase in barrier heights, i.e., we assume $W_{-n}=W_{n}$ and

(26) $$W_{n}=\left\{\begin{array}{cc} F{\left(n-1\right)}^{2} & n\geq 1,\;\;n\;\mathrm{odd}\\ 0 & n\;\mathrm{even}. \end{array}\right.$$

Curve 5 in Figure 3c shows the numerically computed behavior of the survival probability $P\left(t\right)=|a_{0}{\left(t\right)|}^{2}$ for $V_{0}/\kappa =10$ and F=0.1, clearly showing the acceleration of the decay in early stage, with strong revival at longer times.

It should be remarked that decay acceleration mediated by the resonant tunneling effect, observed in all above models, occurs only in the early stage of the dynamics, as shown in Figure 3c. In fact, at long times the survival probability P(t) can become smaller when there are no additional lateral barriers, because the backflow arising from multiple scattering processes and localization effects, responsible for strong non-Markovianity and deviation of the decay from an exponential curve, induce revival effects in the survival probability, which are prevented in the simple two-barrier case.

## 4. Conclusions

The decay of a resonance state trapped in a double potential barrier provides one of the simplest models of unstable quantum systems, which was introduced in a landmark paper by Gamov to explain α decay in nuclear physics. A main question, which has been so far largely overlooked, is whether quantum decay of a metastable state in the double-barrier model can be accelerated by additional lateral barriers. Such additional barriers clearly induce multiple scattering and interference effects, which greatly modify the decay dynamics: the outgoing waves that escape via tunneling from the two barriers can be back-reflected and re-injected into the original spatial region by the later barriers. The resulting decay behavior can strongly deviate from an exponential law and is the result of a complex multiple-interference process which, depending on the choice of the additional barriers, can either decelerate or accelerate the decay. The fact that additional barriers can slow down the decay of the survival probability is not surprising; however, it is more elusive regarding how and why the decay can be accelerated in some cases. In this work, we have shown that a main mechanism that can induce decay acceleration, at least in the early stage of the decay, is resonant tunneling. We have illustrated such a phenomenon by considering in details the decay dynamics of resonant states in tight-binding models, showing that decay acceleration can be observed even when the later barriers are increasingly higher or have some stochastic distribution. The predicted effects could be observable in photonic tunneling experiments using engineered integrated waveguide array circuits.

## Data Availability

No data were generated or analyzed in the presented research.

## Appendix A. Fano–Anderson form of the Quantum Decay on the Lattice

In this Appendix, we derive the canonical Fano–Anderson (or Friedrichs–Lee) form of the quantum decay on the lattice described by the Hamiltonian (18). After letting $|\psi \left(t\right)\rangle =\sum_{n}\psi_{n}\left(t\right)|n\rangle$, the evolution equations for the amplitudes $\psi_{n}\left(t\right)$ read

(A1) $$i\frac{d\psi_{n}}{dt}=-\kappa \left(\psi_{n+1}+\psi_{n-1}\right)+W_{n}\psi_{n}.$$

For the sake of simplicity, we assume that the potential $W_{n}$ is symmetric around n=0, i.e., $W_{-n}=W_{n}$. In this case, for the initial condition $\psi_{n}\left(0\right)=\delta_{n,0}$, the solution to Equation (A1) satisfies the constraint $\psi_{-n}\left(t\right)=\psi_{n}\left(t\right)$, so that we can limit to consider the evolution equations for the amplitudes $\psi_{0}$, $\psi_{1}$, $\psi_{2}$, …which read explicitly

(A2) $$\begin{array}{ccc} i\frac{d\psi_{0}}{dt} & = & -2\kappa \psi_{1} \end{array}$$

(A3) $$\begin{array}{ccc} i\frac{d\psi_{1}}{dt} & = & -\kappa \psi_{0}-\kappa \psi_{2}+V_{0}\psi_{1} \end{array}$$

(A4) $$\begin{array}{ccc} i\frac{d\psi_{2}}{dt} & = & -\kappa \psi_{1}-\kappa \psi_{3} \end{array}$$

(A5) $$\begin{array}{ccc} i\frac{d\psi_{n}}{dt} & = & -\kappa \left(\psi_{n+1}+\psi_{n-1}\right)+W_{n}\psi_{n}\;\;\left(n\geq 3\right). \end{array}$$

In the large $V_{0}/\kappa \gg 1$ limit, we can adiabatically eliminate from the dynamics the amplitude $\psi_{1}$ by assuming $|d\left(\psi_{1}/dt\right)|\ll V_{0}\psi_{1}$ in Equation (A3). This yields

(A6) $$\psi_{1}\left(t\right)≃\frac{\kappa }{V_{0}}\left(\psi_{0}+\psi_{2}\right).$$

After letting

(A7) $$\psi_{0}\left(t\right)=\varphi_{0}\left(t\right)\exp\left(i\kappa^{2}t/V_{0}\right),\;\;\psi_{n}\left(t\right)=\frac{1}{\sqrt{2}}c_{n-1}\exp\left(i\kappa^{2}t/V_{0}\right)\;\;\left(n\geq 2\right)$$

from Equations (A1)–(A7), one obtains

(A8) $$\begin{array}{ccc} i\frac{d\varphi_{0}}{dt} & = & \Omega \varphi_{0}-\kappa_{1}c_{1} \end{array}$$

(A9) $$\begin{array}{ccc} i\frac{dc_{1}}{dt} & = & -\kappa_{1}\varphi_{0}-\kappa c_{2} \end{array}$$

(A10) $$\begin{array}{ccc} i\frac{dc_{n}}{dt} & = & -\kappa \left(c_{n+1}+c_{n-1}\right)+V_{n}c_{n}\;\;\left(n\geq 2\right) \end{array}$$

where we have set

(A11) $$\kappa_{1}\equiv \frac{\sqrt{2}\kappa^{2}}{V_{0}}\;\;,\;\;\Omega \equiv -\frac{\kappa^{2}}{V_{0}}\;\;,\;\;\;V_{n}\equiv W_{n+1}+\frac{\kappa^{2}}{V_{0}}=W_{n+1}-\Omega .$$

To obtain the canonical form of the Fano–Anderson model, let us indicate by

$$u^{\left(\alpha \right)}={\left(u_{1}^{\left(\alpha \right)},u_{2}^{\left(\alpha \right)},u_{3}^{\left(\alpha \right)},\ldots \right)}^{T}$$

and $\omega_{\alpha }$ the eigenvectors and corresponding eigenvalues (energies) of the semi-infinite matrix Hamiltonian H, defined by

(A12) $$H=\left(\begin{array}{cccccccc} 0 & -\kappa & 0 & 0 & 0 & 0 & 0 & \ldots \\ -\kappa & V_{2} & -\kappa & 0 & 0 & 0 & 0 & \ldots \\ 0 & -\kappa & V_{3} & -\kappa & 0 & 0 & 0 & \ldots \\ 0 & 0 & -\kappa & V_{4} & -\kappa & 0 & 0 & \ldots \\ 0 & 0 & 0 & -\kappa & V_{5} & -\kappa & 0 & \ldots \\ \ldots & \ldots & \ldots & \ldots & \ldots & \ldots & \ldots & \ldots \end{array}\right)$$

where α is a discrete index (for localized states) or a continuous variable (for extended states). The eigenstates are assumed to satisfy the orthonormal condition $\langle u^{\left(\alpha \right)}|u^{\left(\beta \right)}\rangle =\delta_{\alpha ,\beta }$ for discrete indices (point spectrum), or $\langle u^{\left(\alpha \right)}|u^{\left(\beta \right)}\rangle =\delta \left(\alpha -\beta \right)$ for continuous indices (continuous spectrum). After expanding the amplitudes $c_{n}\left(t\right)$ as a series (or integral) of the eigenstates of H, i.e., after letting

(A13) $$c_{n}\left(t\right)=\sum_{\alpha }\theta_{\alpha }\left(t\right)u_{n}^{\left(\alpha \right)},$$

from Equations (A8)–(A10) and (A13), one readily obtains the following set of evolution equations:

(A14) $$\begin{array}{ccc} i\frac{d\varphi_{0}}{dt} & = & \Omega \varphi_{0}-\sum_{\alpha }g_{\alpha }\theta_{\alpha } \end{array}$$

(A15) $$\begin{array}{ccc} i\frac{d\theta_{\alpha }}{dt} & = & \omega_{\alpha }\theta_{\alpha }-g_{\alpha }^{*}\varphi_{0} \end{array}$$

where we have set

$$g_{\alpha }\equiv \kappa_{1}u_{1}^{\left(\alpha \right)}.$$

In Equations (A13) and (A14), it is understood that the sum over α should be replaced by an integral over α for the continuous part of the spectrum of H. In their present form, Equations (A14) and (A15) can be derived from the single-level Fano–Anderson (or Friedrichs–Lee) Hamiltonian [24,48,49]

(A16) $${\hat{H}}_{FA}=\Omega |0\rangle \langle 0|+\sum_{\alpha }\omega_{\alpha }\left|\alpha \rangle \langle \alpha \right|-\sum_{\alpha }\left(g_{\alpha }\left|0\rangle \langle \alpha \right|+H.c.\right)$$

which describes the decay of a single level |0〉 of frequency Ω coupled to a discrete or continuous set of states |α〉 (the bath), with frequencies $\omega_{\alpha }$, by a spectral coupling function $g_{\alpha }$. We mention that, in the most general case where the parity condition $W_{-n}=W_{n}$ is not satisfied, one can still obtain a Fano–Anderson Hamiltonian as Equation (A16), but the level |0〉 turns out to be coupled to two baths with different spectral coupling functions.

After eliminating from the dynamics the variables $\theta_{\alpha }\left(t\right)$ by formally solving Equation (A15) with the initial condition $\theta_{\alpha }\left(0\right)=0$ and after letting $\varphi_{0}\left(t\right)=A\left(t\right)\exp\left(-i\Omega t\right)$, from Equation (14) one obtains the following integro-differential equation describing the decay dynamics of the amplitude A(t)

(A17) $$i\frac{dA}{dt}=\int_{0}^{\infty }d\xi G\left(t-\xi \right)A\left(\xi \right)$$

where G(τ) is the memory function, defined by

(A18) $$G\left(\tau \right)=-i\sum_{\alpha }{\left|g_{\alpha }\right|}^{2}\exp\left[-i\left(\omega_{\alpha }-\Omega \right)\tau \right].$$

Only when the single level |0〉 is weakly coupled to a featureless continuum of states with a short memory time $\tau_{m}$—the memory time $\tau_{m}$ is the characteristic decay time of the memory function G(τ)—one can use the Markovian (or Weisskopf–Wigner) approximation [22], and the decay dynamics is well described by an exponential law. In this case, Equation (A18) reduces to a simple differential equation, i.e.,

(A19) $$i\frac{dA}{dt}≃A\left(t\right)\left(\Delta_{R}-i\Delta_{I}\right)$$

where we have set $\Delta_{R}-i\Delta_{I}\equiv \int_{0}^{\infty }d\tau G\left(\tau \right)$ defining the Lamb shift ($\Delta_{R}$) and decay rate ($\Delta_{I}$). After letting $\sum_{\alpha }\to \int d\alpha$ in Equation (A18) and following a standard procedure, the explicit form of the decay rate $\Delta_{I}$ and Lamb shift $\Delta_{R}$ can be calculated as

(A20) $$\Delta_{I}=\pi {\left|g\left(\Omega \right)\right|}^{2}\rho \left(\Omega \right)\;\;,\;\;\;\Delta_{R}=-P\int d\omega \frac{{\left|g\left(\omega \right)\right|}^{2}\rho \left(\omega \right)}{\omega -\Omega }$$

where $g\left(\omega \right)=g_{\alpha =\alpha (\omega )}$ is the spectral coupling function and ρ(ω)≡1/(dω/dα) is the density of states.

As an example of a nearly exponential decay, let us consider the quantum decay in the absence of lateral barriers, i.e., with $W_{n}=0$, corresponding to $V_{n}=-\Omega$ in Equation (A12). In this case, the spectrum of the matrix Hamiltonian H [Equation (A12)] is absolutely continuous, and neglecting the small defect (impurity potential) at the edge, its energy spectrum and corresponding eigenfunctions read (see e.g., [49])

(A21) $$\omega_{\alpha }=-\Omega -2\kappa \cos\alpha \;,\;\;u_{n}^{\left(\alpha \right)}=\sqrt{\frac{2}{\pi }}\sin\left(\alpha n\right)$$

where 0<α<π is a continuous parameter. In this case, the spectral coupling function and density of states are readily calculated as

(A22) $$g\left(\omega \right)=\sqrt{\frac{1}{2\pi }}\frac{\kappa_{1}}{\kappa }\sqrt{4\kappa^{2}-{\left(\omega +\Omega \right)}^{2}}\;,\;\;\rho \left(\omega \right)=\frac{1}{\sqrt{4\kappa^{2}-{\left(\omega +\Omega \right)}^{2}}}.$$

Taking into account that |Ω|≪κ, from Equations (A20) and (A22) one obtains

(A23) $$\Delta_{I}≃\frac{\kappa_{1}^{2}}{\kappa }$$

and thus the lifetime τ reads

(A24) $$\tau =\frac{1}{2\Delta_{I}}≃\frac{\kappa }{2\kappa_{1}^{2}}$$

which is Equation (17) given in the main text.

## Appendix B. Some Qualitative Properties of the Energy Spectrum and Weak Localization

In this Appendix, we present some discussion about the energy spectrum of the tight-binding bipartite Hamiltonian *H* defined by Equation (18) in the main text. After letting ${\left(a_{n}\left(t\right),b_{n}\left(t\right)\right)}^{T}=\left(A,B\right)\exp\left(-iEt\right)$, from Equations (19) and (20) one obtains the spectral problem

(A25) $$\begin{array}{ccc} Ea_{n} & = & -\kappa (b_{n}+b_{n-1}) \end{array}$$

(A26) $$\begin{array}{ccc} Eb_{n} & = & -\kappa \left(a_{n}+a_{n+1}\right)+W_{2n+1}b_{n}. \end{array}$$

It can be readily shown that the energy E=0 belongs to the spectrum of *H* with wave function in one sublattice solely

(A27) $$a_{n}={\left(-1\right)}^{n}\;,\;\;b_{n}=0$$

corresponding to an extended (improper) eigenstate. For E≠0, from Equation (A25) one can express the amplitudes $a_{n}$ in one sublattice in terms of the amplitudes $b_{n}$ in the other sublattice, i.e.,

(A28) $$a_{n}=-\frac{\kappa }{E}\left(b_{n}+b_{n-1}\right).$$

With the substitution of Equation (A28) into Equation (A26), after letting $b_{n}={\left(-1\right)}^{n}\varphi_{n}$, one obtains

(A29) $$-\kappa \left(\varphi_{n+1}+\varphi_{n-1}-2\varphi_{n}\right)+\left(\frac{E}{\kappa }\right)W_{2n+1}\varphi_{n}=\frac{E^{2}}{\kappa }\varphi_{n}.$$

Equation (A29) can be regarded as the spectral problem on a tight-binding lattice in the potential $V_{n}=\left(E/\kappa \right)W_{2n+1}$ with an *energy-dependent* amplitude, vanishing as E→0.

Clearly, if $W_{2n+1}$ is constant, like in the example (i) considered in Section 3, or a periodic function of index *n*, the energy spectrum is absolutely continuous and *H* does not sustain any localized state. In this case, we expect the decay of survival probability to be complete. Conversely, if $W_{2n+1}$ describes some disordered potential, such as the Anderson–Bernoulli model [52] considered in the example (ii) of Section 3, or a deterministic potential with $|W_{2n+1}|$ monotonously increasing and unbounded as |n|→∞, such as the symmetric Stark potential or the parabolic potentials discussed in the examples (iii) and (iv) of Section 3, all eigenstates with E≠0 are strictly speaking localized. This is because any uncorrelated disordered potential of arbitrarily small amplitude in one dimension, or any unbounded potential of small amplitude which is monotonously increasing with |n|, have all eigenstates localized. However, an infinitely countable set of eigenenergies, with weakly extended eigenstates, accumulate toward the zero energy point E=0 of the extended state, with a diverging localization length of corresponding eigenstates. Such a property can be proven rigorously in some special potential models, such as the Stark potential model [53] or other integrable models such as the Maryland model [54]. More generally, for small energies |E/κ|→0, the potential entering in Equation (A5) is almost vanishing and thus we expect that any eigenstate, if localized, should have a large localization length, diverging as E→0. For random potentials, this result, i.e., the divergence of the localization length as E→0, is rigorously proven in [55]. Interestingly, from Equation (A28), it follows that the occupation of the eigenstates with energy close to zero is mostly restricted to one sublattice, namely $|a_{n}\left|\gg \right|b_{n}|$. Such weakly localized eigenstates are nearly resonant with the quasi-bound state localized at site n=0, between the two high barriers. The quasi-bound state can thus couple with such weakly localized states, while other strongly localized states with high energy do not play any main role in the decay dynamics. This means that the decaying quasi-bound state couples to a set of discrete and weakly localized states of the bath, which is responsible rather generally for memory (non-Markovian) effects and strong deviations from an exponential decay law. This explains why the long-time dynamics displays strong revival effects and also limited decay, such as those observed in models (iii), (iv), and (v) discussed in the main text (see Figure 3c). References
